# Social Competence in Infants and Toddlers with Special Health Care Needs: The Roles of Parental Knowledge, Expectations, Attunement, and Attitudes toward Child Independence

**DOI:** 10.3390/children1010005

**Published:** 2014-02-19

**Authors:** Debra Zand, Katherine Pierce, Nicole Thomson, M. Waseem Baig, Cristiana Teodorescu, Sohail Nibras, Rolanda Maxim

**Affiliations:** 1 Knights of Columbus Child Development Center, Cardinal Glennon Children’s Medical Center, Saint Louis University School of Medicine, 3800 Park Avenue, St. Louis, MO 63104, USA; E-Mails: kjpierce@slu.edu (K.P.); mbaig7@slu.edu (M.W.B); cteodore@slu.edu (C.T.); langrisa@slu.edu (S.N.); maximr@slu.edu (R.M.); 2 Department of Psychology, University of Missouri–Saint Louis, 5400 Arsenal, St. Louis, MO 63139, USA; E-Mail: Nicole.Thomson@mimh.edu

**Keywords:** social competence, infant, toddler, attunement, parental knowledge, independence

## Abstract

Little research has empirically addressed the relationships among parental knowledge of child development, parental attunement, parental expectations, and child independence in predicting the social competence of infants and toddlers with special health care needs. We used baseline data from the Strengthening Families Project, a prevention intervention study that tested *Bavolek’s Nurturing Program for Parents and Their Children with Health Challenges* to explore the roles of these variables in predicting social competence in infants and toddlers with special health care needs. Bivariate relationships among the study variables were explored and used to develop and test a model for predicting social competence among these children. Study findings pointed to a combination of indirect and direct influences of parent variables in predicting social competence. Results indicated that parents who encouraged healthy behaviors for developing a sense of power/independence were more likely to have children with social competence developing on schedule. Elements related to parental expectations, however, did not have the hypothesized relationships to social competence. The present study provides preliminary data to support the development of knowledge based interventions. Within medical settings, such interventions may indeed maximize benefit while minimizing cost.

## 1. Introduction

The Maternal and Child Health Bureau (MCHB) defines children with special health care needs (CSHCN) as those “who have or are at increased risk for a chronic physical, developmental, behavioral, or emotional condition and who also require health and related services of a type or amount beyond that which is required by children generally” [1]. A recent population based household survey documented that 10.2 million children in the US (13.9% of all US children) have a special healthcare need, with 20% of all US households with children having at least one CSHCN [2]. Among these children, approximately 41.9% have significant delays in social functioning. Data show that the development of social competence plays a key role in the longitudinal course of a wide array of adaptive outcomes among children, including school readiness and academic success [3,4,5,6]. Although it is well documented that parents play a central role in influencing their child’s developmental trajectories [7], little is known about the causal pathways for enhancing social competence in infants and toddlers with special healthcare needs. The present paper attempted to fill this research gap by investigating the role of parental knowledge, expectations, attunement, and attitudes towards child independence in predicting positive social competence in infants and toddlers with special health care needs. 

### 1.1. Social Competence

Social competence is a complex, multidimensional construct, with some researchers placing greater emphasis on relationships, others on skills, and others on outcomes [8,9,10,11,12,13,14]. Across definitions, common components include pro-social skills, as well as the ability to guide the behaviors of others to meet one’s goals. Additionally, a general consensus exists among developmentalists that expressions of social competence vary with developmental age and build upon previously learned skills and knowledge [15]. For infants and toddlers, operational definitions of social competence have incorporated the presence of a variety of behaviors (e.g., social smile, joint attention, imitation, use of gestures to communicate social intent, pretend play, *etc*.), all which unfold within an interpersonal context of positive ongoing relationships with familiar, nurturing parents [13,16,17]. Consequently, identification of parental qualities that foster social competence has been an important area of inquiry for researchers.

### 1.2. Parental Knowledge

Several studies have documented a positive relationship between parental knowledge of child development (e.g., knowledge of developmental norms and milestones, processes of child development, and caregiving skills) and early childhood outcomes [18,19,20,21,22,23]. In a study of extremely low birth weight infants [21], it was found that maternal knowledge accounted for 13% to 15% of the variance of Bayley scores at eight months of age; with infants of mothers with above-average knowledge scores performing 1 standard deviation (SD) higher than those of parents with average or less than average knowledge. Further support comes from a systematic review of randomized controlled trials of parenting programs which found that increases in maternal knowledge were related to improvements in behavior problems in children [18]. More recent research corroborates these findings, with maternal knowledge of child development being a strong predictor of optimal infant development [24].

Parental knowledge can provide a global cognitive organization for adapting to or anticipating developmental changes in children [25,26]. Research indicates a positive association between parental knowledge and parenting skills [19,25,27], with knowledgeable parents providing more positive attention [23] and responding more sensitively to their child’s cues [28,29]. Taken as a whole, enhanced knowledge of child development may translate into parents being more attuned to their child’s developmental needs, a precursor to infant/toddler social competence.

### 1.3. Parental Expectations

Parental expectations can be conceptualized as parents’ beliefs about the process of child development and the proximal causes of their child’s behavior. Evidence exists showing that parents’ inaccurate beliefs or overestimation of the child’s performance undercuts the child performance [30,31,32]. For instance, mothers with inaccurate expectations behave in a harsher manner towards their child than a mother with more appropriate expectations [33,34,35]. Data also show that parental expectations impact child rearing practices [29]. In their study of maternal expectations of children achieving specific developmental milestones and its relationship to the attainment of social competence, Holloway and Reichhart-Erickson (1989) [36] found a positive relationship between mothers’ social expectations for their children and their children’s level of social competence. Maternal expectations continued to contribute to children’s social competence even after controlling for child-care variables. Other researchers have documented a negative relationship between parental expectations and social competence, linking high expectations to withdrawal behaviors in infants and toddlers [19,37,38]. Finally, research has pointed to the importance of accurate and appropriate parental expectations of child behaviors as a predictor of positive developmental outcomes [19,21].

### 1.4. Parental Attunement

Attunement is a core parenting dimension, which has been theoretically linked to enhanced infant and toddler developmental outcomes [39,40,41,42]. By definition, attuned parents are sensitive to their child’s cues, interpret their meaning accurately, and respond appropriately [43]. Specifically, attunement involves a parent’s ability to understand their child, act on that understanding and adjust to their child’s needs. Attunement has played a central role in various theoretical frameworks including attachment [44,45], sociocultural theories of cognitive development [46,47], and socialization of young children [48,49]. According to Holigrocki and colleagues [39], attunement is fundamental to effective parenting because it helps parents to meet their child’s needs and engage in developmentally appropriate interactions.

Although attunement has been subject to more theorizing than empirical investigation, attunement behaviors have been linked empirically to infant and toddler exploratory behaviors and early social competence [50,51,52,53,54,55]. Several studies have demonstrated positive associations between global measures of parental attunement during free play and children’s exploratory skills [56] and social competence [57]. In their study of infants with varying birth status, Landry and colleagues [52] found that increased maternal responsiveness facilitated growth in social competence. This growth was particularly magnified in infants with low birth weights. 

### 1.5. Parental Attitudes toward Child Independence

Between the ages of one and three, children begin to assert their independence and explore their physical and social environments. This emerging desire for self-sufficiency is essential for the development of social competence. Exploration provides opportunities for play [58], which promotes the development of problem-solving skills and competence in both the intrapersonal and interpersonal realms [59]. According to psycho-developmental theory [60,61], when children are not provided the opportunities to explore and assert themselves, they begin to feel inadequate, and may then become overly dependent upon others. To date, empirical research on child autonomy as a predictor of social competence has focused mainly on parental behaviors within the context of play interactions. Overall, these data have highlighted the detrimental impact of parental over-control on toddler development, with investigators finding a positive relationship between parental over-control and toddler peer inhibition [62,63].

Parental attitudes towards autonomy may also serve as an important predictor of social competence. Across studies, data point to a relationship between parental attitudes and behaviors, with parental attitudes serving an important role in intention to behave [64], a proximal determinant of performance [65]. Indeed, recent research found that a parental attitude towards autonomy was positively correlated with social adjustment among 5-year olds [66,67]. Given the complexity and time consuming nature of caring for children with special health care needs, attitudinal measures may be an easier, less burdensome way of measuring parental support of infant and child independence.

### 1.6. Study Aims

The data from these accumulated studies suggest that the causal relationships among parents’ knowledge of child development, expectations, attunement, and attitudes toward child independence, are complex, involving both direct and indirect effects on child social competence. Based upon the limited empirical research in this area, the current investigation was approached from an exploratory perspective and sought to document the roles of each of these variables in helping explain child social competence among CSHCN. Specific aims of the present article were twofold: (1) to discern the interrelationships among the study variables; and (2) to develop and test a causal model for social/emotional development in CSHCN.

## 2. Method Section

### 2.1. Participants

The present study used baseline data from the Strengthening Families Project, a prevention intervention study that tested Bavolek’s *Nurturing Program for Parents and Their Children with Health Challenges* [68] with parents of children who were enrolled in a Midwestern state’s early intervention system for developmentally at risk or developmentally delayed infants and toddlers, as specified in Part C of the Individuals with Disabilities Education Improvement Act. Of the 154 families referred to the project, a total of 84 participants were enrolled in the study (54.55%). Reasons for refusals included parental medical problems, scheduling conflicts, lack of interest, intervention length, limited free time, and inability to locate parents. Of the families enrolled in the study, 39 (46.4%) had a child who screened positive for having a special healthcare need based on Bethell’s (2002) CSHCN Screener [69]. The CSHCN Screener is a five item tool, which uses non-condition specific, consequences-based criteria to identify children with special health care needs for purposes of quality assessment or other population-based applications [69,70]. To positively screen, a child must meet three conditions: (a) the child currently experiences a specific consequence; (b) the consequence is due to a medical or other health condition; (c) the duration or expected duration of the condition is 12 months or longer [69,70].

All of the participants were female (*N* = 39) with an average age of 31 years (SD = 5.24). Additional demographics are presented in Table 1. The average age of the children was 23 months (SD = 0.77 months). Fifty one-percent were female (*N* = 20). Forty-six percent were premature (*N* = 18), with the average number of weeks of prematurity being 7.72. Diagnoses at time of study entry are located in Table 2, with some children having more than one diagnosis. 

**Table 1:** Demographic Characteristic (total *N* = 39).

Characteristic		
	N	%
Gender		
Females	39	100
Race		
Caucasian	22	56
African-American	16	41
Asian/Pacific Islander	4	3
Marital Status		
Single	19	48.7
Married	15	38.5
Divorced	1	2.6
Unmarried Partners	2	5.1
Separated	2	5.1
Education (years)		
College graduate or more	13	33
Some college	13	33
High school graduate	7	18
Less than high school	6	15
Employment Status		
Employed part-time	8	20
Employed full-time	8	20
Unemployed	23	59
Income		
Under $15,000	11	28.2
$15,001–$25,000	8	20.5
$25,001–$40,000	3	7.7
$40,001–$60,000	5	12.8
$Over $60,000	5	12.8

**Table 2:** Child Diagnoses at Study Entry (*N* = 39).

	% (N)
Premature	46% (*N* = 18)
Autism	15% (*N* = 6)
Neurological Disease	21% (*N* = 8)
Deaf	8% (*N* = 3)
Down Syndrome	5% (*N* = 2)
Cerebral Palsy	5% (*N* = 2)
Cleft Palate	5% (*N* = 2)
CMV	3% (*N* = 1)
Muscle Tone Deficits	3% (*N* = 1)
Blind	3% (*N* = 1)
Skeletal Dysplasia	3% (*N* = 1)

### 2.2. Design and Procedures

An experimental, repeated measures design was employed. However, only baseline data is reported here. Prior to the collection of data, all study staff were trained on human subjects’ procedures and human subjects’ approval was secured. After obtaining parental written permission, case managers in the early intervention system referred families to study staff. At the time of referral, the research team contacted the parents and scheduled an appointment to secure informed written consent. Once consent was secured, parents were randomized into one of two conditions: (a) Intervention Group—Services as Usual + Nurturing Parenting and (b) Control Group—Services-As-Usual. Within Missouri’s early intervention system, services as usual consist of an array of individualized interventions that focus on the needs of the child and stress the importance of enhancing family capacity to meet these needs [71]. In the present study, the *Nurturing Parenting* program augmented services as usual by providing parents with eight 2hour group sessions one day a week for eight weeks that focused on teaching age-appropriate expectations and neurological development of children, developing empathy and self-worth in parents, using nurturing, non-violent strategies and techniques in establishing family discipline; and empowering parents to utilize their personal power to make healthy choices [72,73]. Data were collected at baseline and eight weeks-post baseline. Data collection was proctored by trained research assistants and immediately entered into a laptop. At the time of survey administration, all participants were instructed to complete the tools on the study’s index child. The index child was the child receiving early intervention services from age 0–3.

### 2.3. Measures

The *Adult-Adolescent Parenting Inventory-Version 2 (AAPI-2*) [74] is a 40-item inventory with strong psychometric properties designed to assess the parenting and child rearing attitudes of adolescent and adult parent (or pre-parent) populations. Lower scores indicate higher risk for abuse. All items are presented on a 5-point Likert scale of Strongly Agree, Agree, Disagree, Strongly Disagree, and Uncertain. It has been assessed at the 5th grade reading level. The measure results in an index risk of five specific parenting and child rearing behaviors: (1) Expectations; (2) Attunement; (3) Discipline; (4) Roles; and, (5) Power and Independence. The present study used the Expectations, Attunement, and Power/Independence subscales. The Expectations subscale is comprised of seven items. Examples include: “Strong willed children must be taught to mind their parents,” and “Parents need to push their children to do better.” The Attunement subscale consists of 10 items. Sample items include: “Children should keep their feelings to themselves,” and “Children cry just to get attention.” The Power/Independence subscale consists of five items, including “Children need to be allowed freedom to explore their world in safety,” and “Children should be potty trained when they are ready and not before.” Cronbach’s alphas for the Expectations, Attunement, and Power/Independence subscales were 0.83, 0.83, and 0.80, respectively. 

The *Knowledge of Infant Development Inventory (KIDI)* [75] is a 75-item instrument designed to obtain comprehensive information on parents’ factual knowledge of child rearing practices, developmental processes, and infant norms of behavior. It samples physical, social, linguistic, perceptual, and cognitive domains of infant development and includes items concerning early experience, learning, social influences, atypical development, individual differences, and health and safety practices. For the present study, the total scale score was used (Cronbach’s alpha = 0.89). Sample items include: “A 1-year old knows right from wrong,” “If a baby is shy or fussy that usually means there is an emotional problem,” and “Most two-year-olds can tell the difference between a make-believe story on TV and a true one.”

The *Ages and Stages Questionnaire-Third Edition (ASQ-3)* [17,76] is a comprehensive developmental assessment, with strong psychometric properties appropriate for children as young as one month old through five years. Parents respond to items on the questionnaire using a 3-point scale: “yes” my child exhibits that behavior or skill, “sometimes” my child exhibits that behavior or skill, or my child does “not yet” exhibit that behavior or skill. Questionnaires take approximately 10–15 min to complete. A cutoff score is calculated for five developmental domains (communication, gross motor skills, fine motor skills, problem-solving, and personal-social skills) indicating a need for further assessment or developing on schedule. Subscales can be administered and interpreted alone. Although the domains remain the same across age groups, subscale questions vary with child age. The present study used the personal-social subscale. Cronbach’s alphas for the personal social skills subscale ranged from 0.58 to 0.85, depending upon the form used. Sample items from the 22 month questionnaire include: “When playing with either a stuffed animal or a doll, does your child pretend to rock it, feed it, change its diapers, put it to bed, and so forth?” and “Does your child copy the activities you do, such as wipe up a spill, sweep, shave, or comb hair?”

## 3. Results and Discussion

### 3.1. Preliminary Analyses: Step A

Chi-square analyses and t-tests of key baseline characteristics (child age, parental age, race, parental education, parental gender, child gender, and child diagnosis) revealed no statistically significant differences between intervention group participants and control group participants. Consequently, for the present study, baseline data from both groups were pooled for data analytic purposes. 

### 3.2. Descriptives and Correlations: Step B

Significant positive relationships were found between parental knowledge and expectations, knowledge and attunement, attunement and child power/independence, and child power/independence and child social competence (see Table 3).

**Table 3:** Correlations and Descriptive Statistics for Knowledge, Expectations, Attunement, Power/Independence, and Social Competence Variables (*N* = 39).

	1	2	3	4	5
1. Knowledge	1.0	0.46 ^**^	0.55 ^***^	0.12	0.07
2. Expectations		1.0	0.77	0.29	0.26
3. Attunement			1.0	0.42 ^**^	0.27
4. Power/Independence				1.0	0.40 ^**^
5. Social Competence					1.0
Mean	0.65	2.87	3.87	2.89	NA
*Standard Deviation*	0.16	0.68	0.63	0.52	NA

^**^ *p* ≤ 0.01, ^***^ *p* ≤ 0.001.

### 3.3. Model Development and Path Analysis: Step C

**Figure 1:**
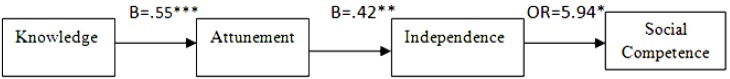
Results of path analysis for social competence. *Note:* For linear regression of knowledge of infant development on parental attunement R^2^ = 0.30 (R^2^*_adj_* = 0.28), *p* ≤ 0.001. For linear regression of parental attunement on power/independence R^2^ = 0.17 (R^2^*_adj_* = 0.15), *p* ≤ 0.01. For logistic regression of power/independence on social/emotional cutoff (binary) Nagelkerke R^2^ = 0.19, *p* ≤ 0.05; odds ratio: (OR) = 5.94, *p* ≤ 0.05); * *p* ≤ 0.05, ** *p* ≤ 0.01, *** *p* ≤ 0.001.

Based on the correlational analyses, a path analysis using the method described by Kerlinger (1973) was completed using linear regression analyses as the path analytic strategy [77]. As seen in Figure 1, participants who scored high on knowledge were also more likely to score high on attunement R^2^ = 0.30, *p* = 0.001. Those who scored high on attunement also tended to score high on child power/independence, R^2^ = 0.17, *p* = 0.008). 

Because social competence was a categorical outcome, logistic regression was used to examine the association between power/independence and social competence. Results indicated that compared with those who needed further assessment, those children developing on schedule were six times more likely to have parents who encouraged healthy behaviors for developing a sense of power/independence (OR = 5.94, *p* < 0.05). Elements related to parental expectations, however, did not have the hypothesized relationships to social competence (expectations OR = 2.7, *p* = 0.12).

### 3.4. Discussion

The present study explored the roles of parental knowledge, expectations, attunement, and attitudes towards child independence in predicting social competence in infants and toddlers with special health care needs. Bivariate relationships among the study variables were explored and used to develop and test a model for predicting social competence among these children. Study findings pointed to a combination of indirect and direct influences of parent variables in predicting social competence. Parental knowledge of child development and attunement were indirectly related to social competence. Specifically, parents who displayed greater knowledge of child development were more likely to report being attuned with their child. Greater attunement predicted positive attitudes towards child independence, which then predicted social competence. These data lend support to theories that emphasize the importance of parental knowledge as precursor to positive developmental outcomes. Such knowledge may provide a cognitive schema for informing parental behaviors that foster positive developmental trajectories.

For parents of children with special health care needs, provision of information regarding normative development may be particularly important. By definition, such children are at high risk for developmental delays. Accurate assessment of developmental delay is predicated on understanding normative behaviors. Several studies indicate that, overall, parents have limited knowledge of typical child development, and have inaccurate expectations regarding age-appropriate behaviors [21,23,78,79,80,81]. For parents of infants and toddlers with special health care needs this lack of general knowledge may impede their ability to discern normative development from delayed development, and thereby negatively impact the accuracy by which they interpret their child’s cues and respond appropriately [82,83,84]. Accurate knowledge of child development may help parents to read their child’s signals and appropriately support their child’s natural curiosity and exploratory behaviors; both of which are considered essential precursors to the development of social competence.

### 3.5. Limitations and Future Directions

The present study did not seek to identify all factors predictive of social competence in infants and toddlers with special health care needs, but instead to serve only as a starting point for developing a causal model. Within the developmental literature, data suggest that social competence is multi-determined and contextually driven [37,85,86,87,88,89]. A recent study found that a complex interaction between specific individual-level toddler risk factors and maternal parenting behavior predicted social competence among children transitioning into kindergarten [85]. Differences in predictors of social competence across cultures have also been documented [86]. Although it is unclear whether these findings generalize to infants and toddlers with special healthcare needs, they do illustrate the need for a more comprehensive understanding of factors that influence social competence among this population.

It is also important to keep in mind that these data do not represent all infants and toddlers with special health care needs. This study employed a relatively small sample of parents who had volunteered to enroll in an early childhood prevention intervention study designed to foster nurturing parenting skills. Future research is needed to investigate variables that impact infant and toddler social competence in a larger, more representative sample of parents of infants and toddlers with special health care needs. A larger sample also would enable researchers to assess model fit through use of advanced statistics such as structural equation modeling.

Finally, the present study employed self-report, single-informant tools with moderate to high internal consistency reliabilities. The sole reliance on parent self-report may not permit researchers and practitioners to consider alternative explanations for social competence. Certainly, a multi-method, multi-source assessment of factors related to infant and toddler social competence would provide a more comprehensive picture for interventionists who wish to design programs to meet the developmental needs of these children; especially if they include observation measures of parent-child interactions in natural settings. Additionally, although adequate for conducting exploratory research, some of the current study’s internal consistency estimates are considered low for instruments used to make decisions about individuals [90]. Consequently, it will be important for these findings to be replicated prior to applying them in real world settings.

## 4. Conclusions

The findings of this study have implications for families and health providers. Children with special healthcare needs use health and related services of a type or amount beyond that required by children in the general population [1], placing tremendous burden on the child, family, and general health service delivery system [91,92,93,94,95]. To help reduce burden, brief effective interventions that promote positive developmental trajectories are needed. The present study provides preliminary data to support the development of knowledge based interventions. Within medical settings, such interventions may indeed maximize benefit while minimizing cost.
